# Effects of Increasing Seawater Carbon Dioxide Concentrations on Chain Formation of the Diatom *Asterionellopsis glacialis*

**DOI:** 10.1371/journal.pone.0090749

**Published:** 2014-03-11

**Authors:** Joana Barcelos e Ramos, Kai Georg Schulz, Colin Brownlee, Scarlett Sett, Eduardo Brito Azevedo

**Affiliations:** 1 Centre of Climate, Meteorology and Global Change of the University of the Azores and the Research Centre for Agricultural and Environmental Science and Technology of the Azores, Angra do Heroísmo, Azores, Portugal; 2 Centre for Coastal Biogeochemistry, School of Environmental Science and Management, Southern Cross University, Lismore, Australia; 3 The Marine Biological Association of the United Kingdom, Plymouth, Devon, United Kingdom; 4 GEOMAR | Helmholtz Centre for Ocean Research Kiel, Kiel, Germany; Scottish Association for Marine Science, United Kingdom

## Abstract

Diatoms can occur as single cells or as chain-forming aggregates. These two strategies affect buoyancy, predator evasion, light absorption and nutrient uptake. Adjacent cells in chains establish connections through various processes that determine strength and flexibility of the bonds, and at distinct cellular locations defining colony structure. Chain length has been found to vary with temperature and nutrient availability as well as being positively correlated with growth rate. However, the potential effect of enhanced carbon dioxide (CO_2_) concentrations and consequent changes in seawater carbonate chemistry on chain formation is virtually unknown. Here we report on experiments with semi-continuous cultures of the freshly isolated diatom *Asterionellopsis glacialis* grown under increasing CO_2_ levels ranging from 320 to 3400 µatm. We show that the number of cells comprising a chain, and therefore chain length, increases with rising CO_2_ concentrations. We also demonstrate that while cell division rate changes with CO_2_ concentrations, carbon, nitrogen and phosphorus cellular quotas vary proportionally, evident by unchanged organic matter ratios. Finally, beyond the optimum CO_2_ concentration for growth, carbon allocation changes from cellular storage to increased exudation of dissolved organic carbon. The observed structural adjustment in colony size could enable growth at high CO_2_ levels, since longer, spiral-shaped chains are likely to create microclimates with higher pH during the light period. Moreover increased chain length of *Asterionellopsis glacialis* may influence buoyancy and, consequently, affect competitive fitness as well as sinking rates. This would potentially impact the delicate balance between the microbial loop and export of organic matter, with consequences for atmospheric carbon dioxide.

## Introduction

Amongst the most recent (180 Ma [1]) planktonic unicellular autotrophs of Earth's Oceans, diatoms exhibit diverse morphologies and ecological strategies. For instance, diatoms can occur either as single cells or as colonies, influencing buoyancy, predator evasion, light absorption and nutrient uptake. The processes by which adjacent cells in chains establish connections determine strength and flexibility of the bonds, and their distinct cellular locations define colony structure. In a constantly changing environment cells, whether solitary or in colonies, need to be able to regulate their gene expression, physiology and signalling. Colonial species such as the diatom *Skeletonema costatum* (*S. costatum*), have been shown to alter chain formation, namely by increasing chain length with temperature (from 6 to 17°C) and nutrient availability [2]. Finaly, chain length has been found to be positively correlated with growth rates [2] and to follow the inverse trend in senescent populations [3]–[4].

Adjacent cells in a chain of *S. costatum* attach by external silica tubes at the margin of the valves ([4], http://www.protistcentral.org/index.php/Taxa/get/taxa_id/2843). However, this is not a unique strategy and other species establish cell-cell connections by means of mucus, bands or even septa fusion [5]. In the case of the cosmopolitan *Asterionellopsis glacialis* (*A. glacialis*), cells attach at the valve apices by exuded polysaccharides which form mucilage pads [1]. Changes in seawater chemistry could influence the binding strength or secretion of polysaccharides, especially when charged, and potentially affect chain formation. This in turn may influence buoyancy, predator or pathogen evasion, light absorption and nutrient uptake ([6], summarized in Beardall et al. [7]). Furthermore, cells in a chain such as in spirals of *A. glacialis* may develop a microenvironment with lower CO_2_ concentrations/higher pH in the centre of the colony during daylight where photosynthetic removal of CO_2_ can lead to diffusional limitation and localized depletion. However, virtually nothing is known about the effects of varying environmental conditions (e.g. pH) on chain length of *A. glacialis*.

Atmospheric CO_2_ has been increasing since the industrial era, reaching values (currently ∼400 µatm) above those observed in the last 800 000 years (from ∼180 to ∼280 µatm). In a business as usual scenario [8] CO_2_ is projected to continue to increase, reaching about 750 µatm by the year 2100. As CO_2_ increases in the atmosphere, it also enters the ocean by air-sea gas exchange, increasing its average concentration and shifting the carbonate chemistry to a more acidic environment (termed ocean acidification).

Recent studies have revealed that changes in carbonate chemistry as expected in the future ocean [8] can affect marine phytoplankton in various ways (e.g. [9]–[10]). Until now, studies with diatoms mostly focussed on carbon acquisition [11], [12] or found higher growth, carbon fixation rates and/or increased efficiency of energy conversion to photosynthesis [13]–[17] under high CO_2_ concentrations. Considering that these silica shielded planktonic primary producers are thought to account for up to 45% of net primary productivity in the ocean [18], a further increase in carbon fixation could act as negative feedback for atmospheric CO_2_. However, the response of diatoms to increasing CO_2_ is still poorly understood and the importance of organization strategies has been mostly overlooked so far [19]. Here we investigate whether changing seawater carbonate chemistry affects the physiology (cell division and organic matter production rates and element stoichiometry) and colony/chain formation of the cosmopolitan diatom *A. glacialis*. Additionally, we provide reasoning that the observed response of *A. glacialis* is driven by distinct parameters of the carbonate system (carbonation versus pH) depending on the CO_2_ concentration to which the cells were being exposed.

## Materials and Methods

### Experimental Setup

Freshly isolated monospecific cultures of the cosmopolitan *Asterionellopsis glacialis* (strain isolated offshore the Azores (CCMMG_1, October 2011)) were grown semi-continuously under varying CO_2_ levels (between approximately 320 and 3400 µatm, pH_total scale_ of ∼8.15 to 7.24, for more detail see Table 1) for a minimum of 20 generations before the start of the experiments. No specific permissions were required for these location (38°37′N27°15′W)/activities at the time of collection. The field studies did not involve endangered or protected species. All cultures were grown in 0.2 µm sterile filtered North Atlantic water (salinity of 36) enriched with approximately 4 µmol l^−1^ phosphate and 64 µmol l^−1^ of nitrate and silicate (the increase of total alkalinity upon addition of Na_2_SiO_3_ was compensated for by HCl addition), and with trace metals and vitamins following f/8 [20] at 20°C, a photon flux density of 220 µmol m^−2^ s^−1^ (supplied from OSRAM L 18W/840, Lumilux, coolwhite) and a 14/10 h light/dark cycle.

**Table 1:** Carbonate chemistry at the beginning, end and through (average) the experiments.

Culture	Treatment	*p*CO_2_ (µatm)	Avg *p*CO_2_ (µatm)	TA (µmol kg-1)	pHt	HCO_3_^−^ (µmol kg-1)	CO_3_^2−^ (µmol kg-1)	CO_2_ (µmol kg-1)	DIC (µmol kg-1)
Initial	1	426		2370	8.030	1899	188	13.7	2100.44
	2	786		2364	7.799	2062	120	25.3	2207.40
	3	1709		2361	7.490	2201	63	54.9	2318.92
	4	4637		2351	7.073	2284	25	149.0	2457.91
Final	1	216	321	2397	8.270	1678	289	6.9	1973.72
	1	320	373	2302	8.121	1761	215	10.3	1986.71
	1	329	377	2385	8.125	1825	225	10.6	2059.96
	1	331	378	2365	8.120	1814	221	10.6	2045.79
	2	329	558	2392	8.126	1831	226	10.6	2068.19
	2	329	558	2386	8.125	1827	225	10.6	2062.94
	2	550	668	2370	7.936	1977	158	17.7	2151.83
	2	561	674	2366	7.927	1978	155	18.0	2150.59
	2	587	687	2370	7.911	1994	150	18.9	2162.75
	3	815	1262	2369	7.787	2075	117	26.2	2218.75
	3	855	1282	2368	7.767	2085	113	27.5	2225.04
	3	1186	1447	2357	7.637	2141	86	38.1	2264.52
	3	1191	1450	2353	7.635	2138	85	38.3	2261.76
	3	1241	1475	2367	7.621	2157	83	39.9	2279.91
	4	2052	3345	2360	7.415	2224	53	65.9	2343.59
	4	2072	3354	2359	7.411	2224	53	66.6	2343.96
	4	2145	3391	2360	7.397	2229	51	68.9	2348.80

pCO_2_, HCO_3_^−^, CO_3_^2−^, CO_2_ and dissolved inorganic carbon (DIC) were calculated based on TA and pH using CO2sys.

The media was acclimated to the temperature of the experiment before inoculation. Cell densities varied on average between 140 and 16000 cell ml^−1^, therefore minimizing changes in seawater carbonate chemistry (average DIC drawdown of 3.9%). All cultures were vertically rotated (10 times gently) daily one hour after the beginning of the light phase to avoid aggregation, sedimentation and self-shading during the light phase.

### Carbonate system

The carbonate system was manipulated by adding calculated amounts of NaHCO_3_ and HCl in a closed system following Schulz et al. [21]. Alkalinity was measured by potentiometric titration following Dickson et al. [22], using a Metrohm Titrino Plus 848 equipped with a 869 Compact Sample Changer, and calibrated with certified reference material supplied by A. Dickson. The pH was measured using a glass electrode (WTW, pH 340i) and calibrated with a TRIS seawater buffer, supplied by A. Dickson.

Carbonate chemistry was calculated from measured temperature, salinity, silicate and phosphate concentrations, and pH and TA using CO2sys [23], with the equilibrium constants determined by Mehrbach et al. [24] as refitted by Dickson and Millero [25].

### Nutrients

Samples for the determination of nutrients at the start and end of incubations were filtered through a polyethersulfone (PES) 0.2 µm syringe filter and stored at −20°C until being analysed. Concentrations of nitrate, silicate and phosphate were measured following Hansen and Koroleff [26], by means of a spectrophotometer (Cary 50 Probe, Varian).

### Cellular element quotas and dissolved organic carbon exudation

Samples for cellular particulate organic carbon (POC), nitrogen (PON) and phosphorus (POP) were gently filtered (200 mbar) through pre-combusted GF/F filters (6 h, 450°C) and stored at −20°C until analyses. POC and PON samples were then dried (4 h, 60°C), packed in tin boats and analysed in a gas chromatograph (EURO EA Elemental Analyser, EUROVECTOR equipped with a thermal conductivity detector and an element analyzer) following Sharp [27]. POP filters were oxidized by potassium peroxydisulphate to dissolved inorganic phosphorus and measured colorimetrically by means of a spectrophotometer (UV-1202, UV-VIS Spectrophotometer, SHIMADZU) following Hansen and Koroleff [28]. Daily production rates were calculated by multiplying cellular quotas (POC, PON, POP per cell abundances) with the corresponding cell division rates μ (see below).

Dissolved organic carbon was estimated as the difference between calculated (from TA and pH) inorganic carbon consumption (ΔDIC) and net build-up of organic matter (ΔPOC).

### Cell numbers and growth rates

Cell abundances (on average ∼800 cells per sample were counted) and the number of cells in a chain were determined from samples fixed with Lugol (2% final concentrations) by means of an inverted microscope (Leica DMIL) at 200× magnification. Cell division rate (μ) was calculated as:
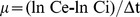
(1)where Ce and Ci refer to end and initial respectively of concentrations of cells, POP, POC or PON, and Δt to the duration of the incubation period in days.

The equation used for fitting cell division rates (μ_tl_, tendency line) based on cell numbers and on all parameters (cell concentrations, POP, POC and PON) followed a modified Michaelis Menten kinetic [29], allowing for optimum curve characteristics, as:
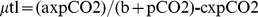
(2)in which **a** (cell 3.35, all parameters 3.46) and **b** (cell 89.6, all parameters 93.21) are random fitting parameters, **c** (cell 0.0006739, all parameters 0.0006819) describes the CO_2_ sensitivity and *p*CO_2_ (µatm) refers to the CO_2_ level.

### Statistical analysis

Statistical significance of the data was tested for by Anova (significance determined as 99%, p<0.01), using the program R.

## Results

**Figure 1:**
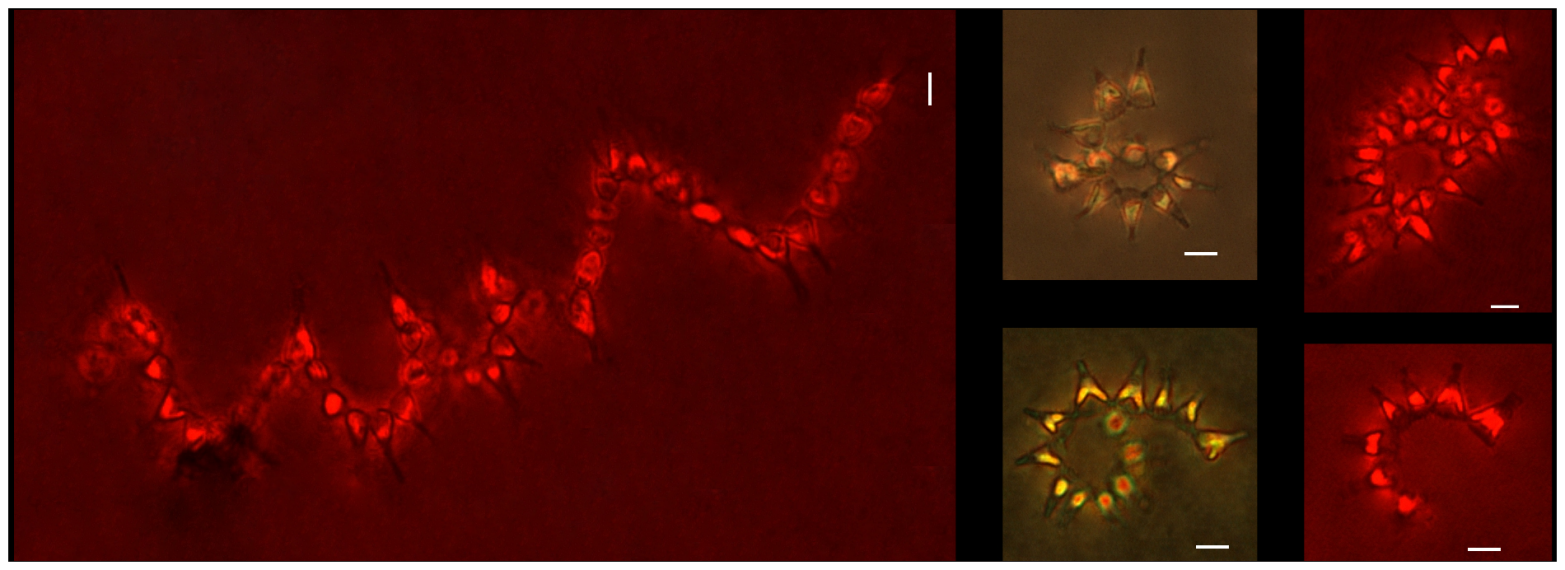
Chain disposition of *Asterionellopsis glacialis* visualized at 200× magnification with an inverted microcope (Leica DMIL). The photographs chosen are representative of chains of different lengths irrespective of the carbon dioxide concentration (photos in red show auto-fluorescence achieved by using the filter N2.1 green). Note the proximity between cells in the spirals. Scale bars correspond to 10 µm.

**Figure 2:**
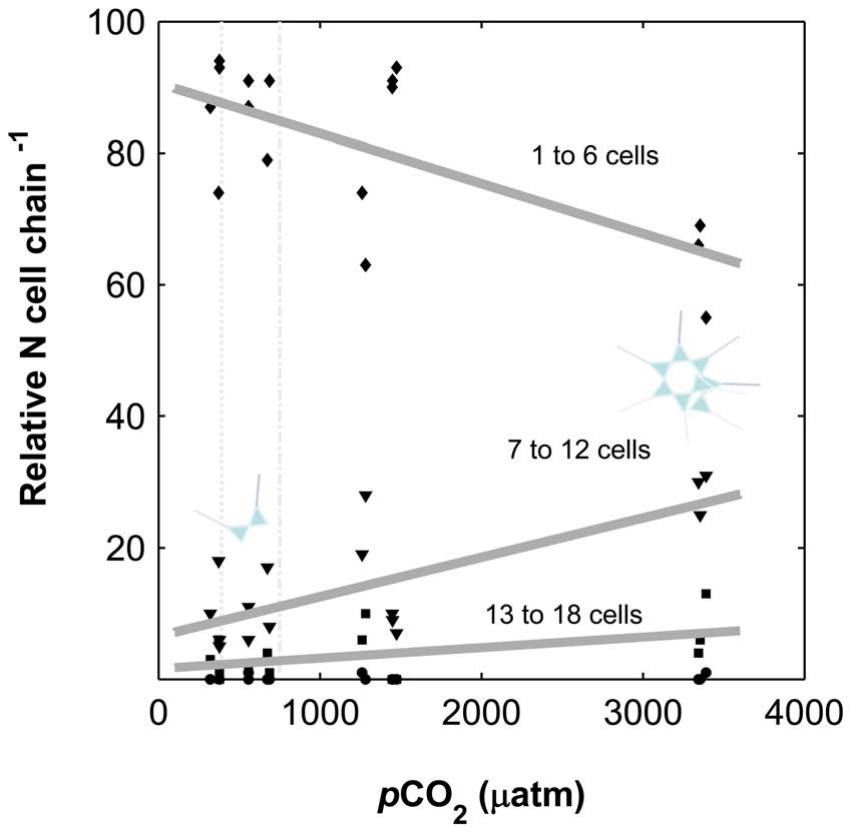
Relative number of cells per chain at increasing carbon dioxide levels (*p*CO_2_). 1 to 6 cells in one chain (diamonds, p<0.01), 7 to 12 cells in one chain (triangles, p<0.01), 13 to 18 cells in one chain (squares, p = 0.05), more than 19 cells in one chain (circles). Solid lines correspond to a linear fit of all data points from each size class. Dashed vertical lines correspond to 390 and 750 µatm. Schemes of colonies represent the increase of longer chains with increasing carbon dioxide.

When CO_2_ was increased from approximately 320 to 3400 µatm, the relative number of chains composed of 1 to 6 cells decreased (p<0.01) while longer chains with 7 to 18 cells increased (7 to 12 cells p<0.01 and 13 to 18 cells p = 0.05, i.e. significant at a 95% confidence level) (Fig. 1, Fig. 2). Data was fitted linearly.

**Figure 3:**
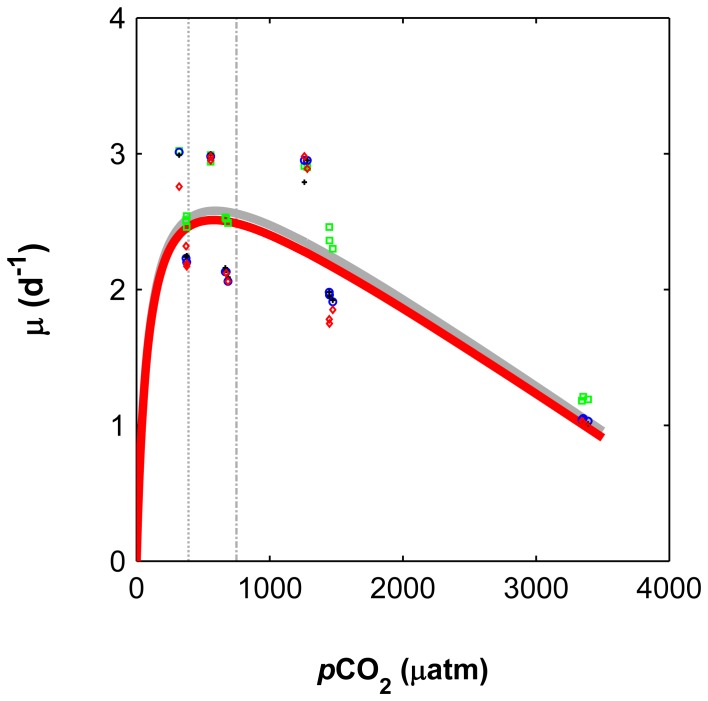
Cell division rates based on cell counts and POP/C/N in relation to CO_2_ levels (from ∼600 to 3400 µatm, p<0.01). The solid line depicts a tendency line obtained by fitting the *Asterionellopsis glacialis* cell based data (red line) and a combination of cell, POP, POC and PON data (grey line) to an equation following a modified Michaelis-Menten curve. Markers correspond to cell division rates based on POP (green), POC (black), PON (blue) and cell numbers (red). Dashed vertical lines correspond to 390 and 750 µatm.

**Figure 4:**
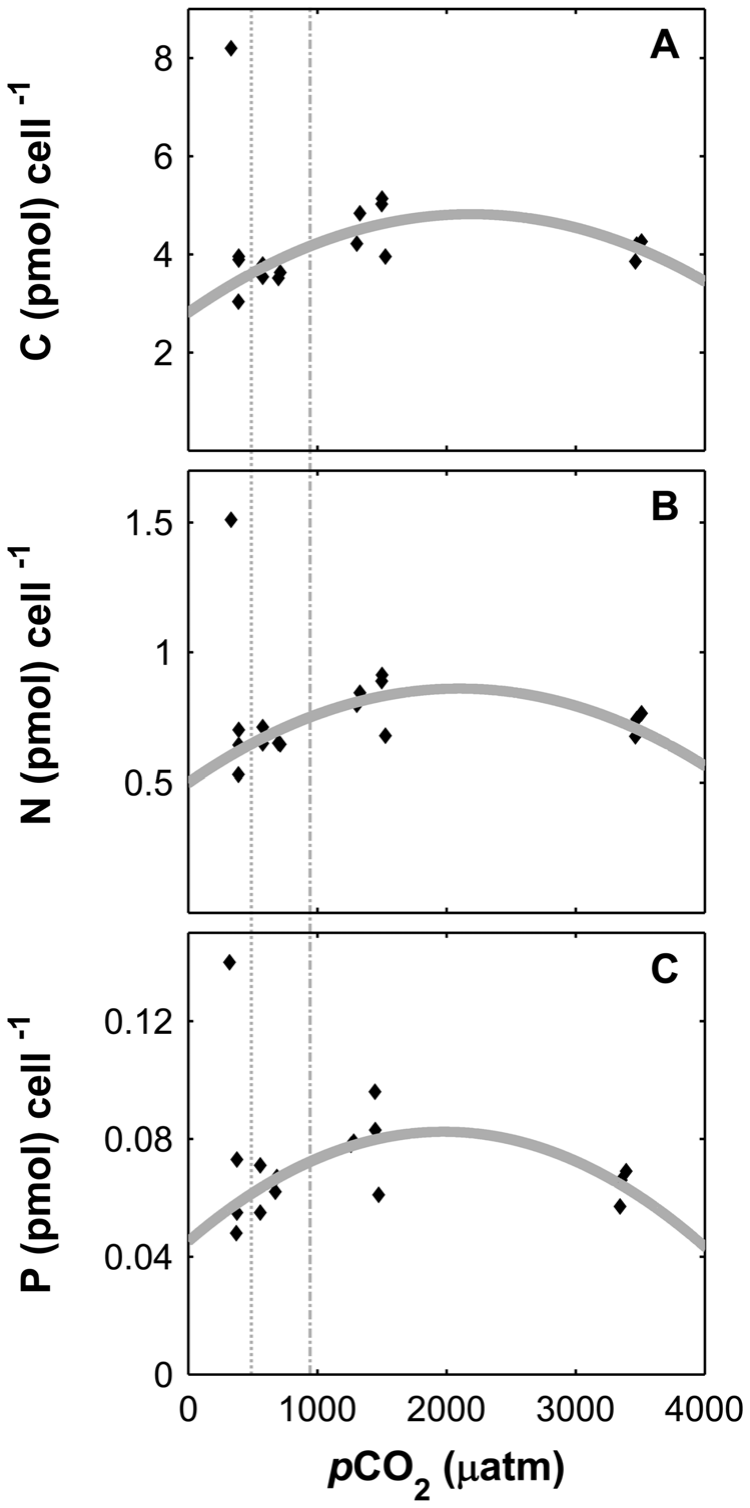
Cellular element quotas of *Asterionellopsis glacialis* at increasing CO_2_ levels (*p*CO_2_). Carbon (A), nitrogen (B) and phosphorus (C). Lines denote a polynomial fit of the respective data (cellular POC: y = −4.18×10^−7^a^2^+0.002a+2.81; PON: y = −8.22×10^−8^a^2^+3.45×10^−4^a+0.50; POP: y = −9.54×10^−9^a^2^+3.77×10^−5^a+0.045, without considering the outlier value correspondent to 320 µatm). Dashed vertical lines correspond to 390 and 750 µatm.

**Figure 5:**
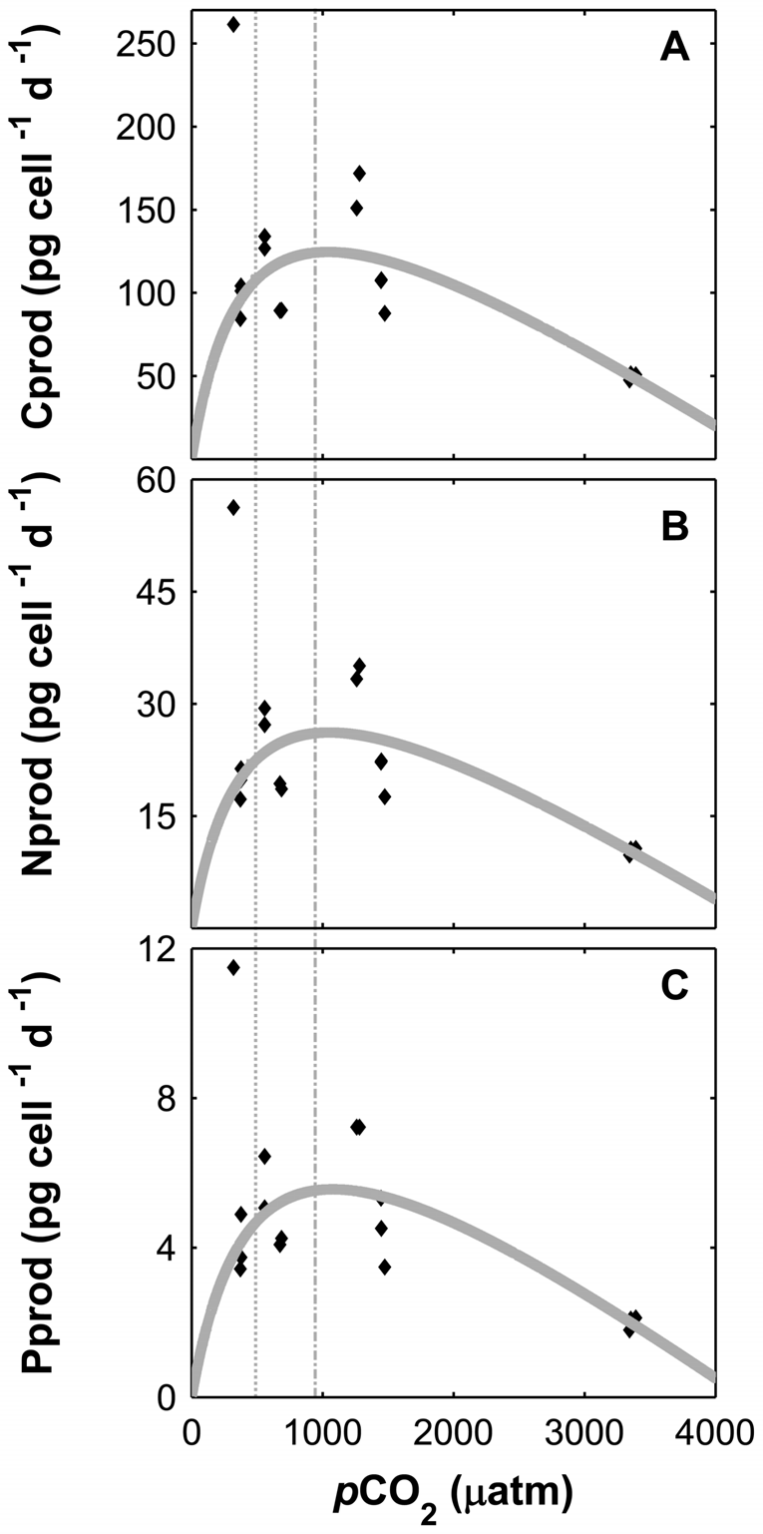
Organic matter production rates of *Asterionellopsis glacialis* under increasing CO_2_ levels (*p*CO_2_). Carbon(A), nitrogen (B) and phosphorus (C). Lines denote a modified Michaelis Menten kinetic of the respective data (without considering the outlier value correspondent to 320 µatm). Dashed vertical lines correspond to 390 and 750 µatm.

Cell division rates based on cell numbers and organic matter (POC, PON and POP) followed a modified modified Michaelis Menten curve (R^2^ all data = 0.69), not varying significantly from 320 to 600 µatm, but decreasing with rising CO_2_ between ∼600 to 3400 µatm (Fig. 3). In fact, cell division rates based on cell numbers decreased on average 2.3 fold (p<0.01) with higher CO_2_ between the interval considered (∼600 to 3400 µatm). For CO_2_ levels ranging from ∼600 to 1470 µatm, the decrease was associated with an increase by approximately 1.5 fold of the cellular quotas of carbon (C), nitrogen (N), and phosphorus (P) (Fig. 4). This trend was not maintained at CO_2_ levels higher than 1470 µatm, at which C, N and P quotas decreased. A similar trend, but now following a modified Michaelis Menten kinetic, to cellular contents was observed for N, C and P production rates (Fig. 5).

**Figure 6:**
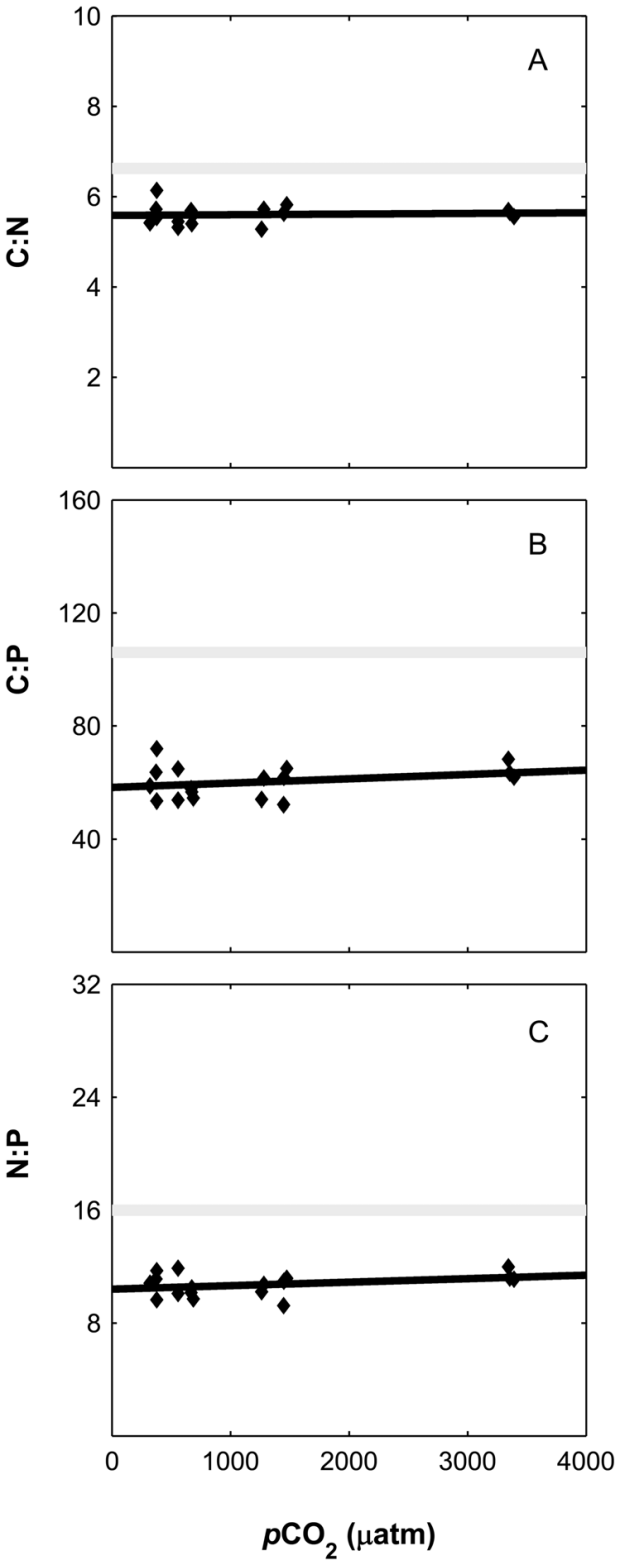
Particulate organic matter ratios (µmol/µmol) of *Asterionellopsis glacialis* under increasing CO_2_ levels (*p*CO_2_). Carbon to nitrogen (A), carbon to phosphorus (B) and nitrogen to phosphorus (C). The solid black line was obtained by fitting the data linearly. Grey line denotes the Redfield value.

**Figure 7:**
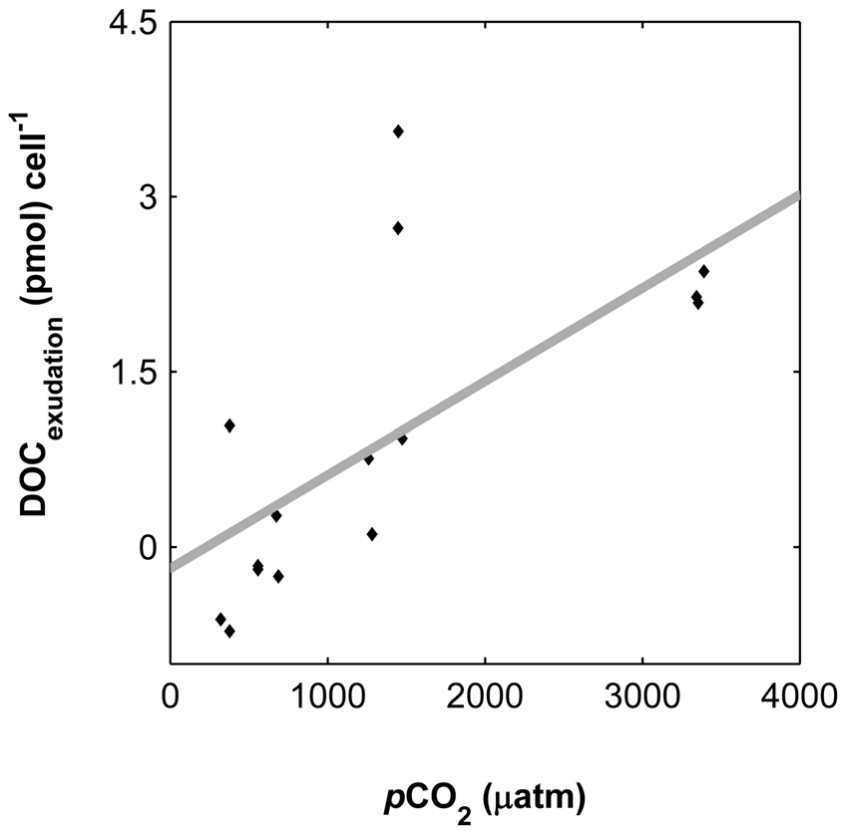
Cellular net exudation with increasing CO_2_ (*p*CO_2_). Exudation calculated here as the difference between calculated inorganic carbon consumption (ΔDIC) and particulate organic carbon build-up (ΔPOC) in relation to CO_2_ levels (*p*CO_2_) per cell (p<0.01). Solid line was obtained by linearly fitting the data.

Despite the observed trends in cellular quotas and production rates no significant correlation was obtained for organic element ratios (C to N, C to P and N to P), showing a proportional storage at all CO_2_ levels tested (Fig. 6). Finally, associated with the decrease in cell division rate, there was a ∼2 fold increase (p<0.01) of exudation of carbon in the form of dissolved organic carbon (DOC) as depicted in the linear increasing difference between calculated (from TA and pH, except for one value since TA was not precise) inorganic carbon consumption (ΔDIC) and net build-up of organic matter (ΔPOC) from 1260 to 3400 µatm (range of CO_2_ levels corresponding to positive values of exudation). This trend could be pinpointed to increased cellular exudation (Fig. 7) and was maintained also as cellular exudation rates (data not shown).

## Discussion

### Growth response to enhanced CO_2_/decreased pH

Despite the importance of diatoms in the marine carbon and silica cycles only a few studies have considered the effects of varying CO_2_ concentrations on their physiology. Indeed, diatoms are thought to be less sensitive to increasing CO_2_ than other phytoplankton groups such as coccolithophores. A number of studies have analysed the influence of CO_2_ levels on diatom carbon concentration mechanisms (e.g. [12], [30], [31], [32], [33]). Nevertheless, there are only a few studies directly addressing the potential effects of enhanced CO_2_ levels on diatoms, with the majority showing null to little effects (*Thalassiosira weissflogii* under 36 to 1800 ppmv [12]; *Thalassiosira pseudonana* under 380 and 760 ppmv [34] and *Phaeodactylum tricornutum* from ∼20 to 800 ppmv [17]) or positive effects (e.g. enhanced growth rate, carbon fixation and/or increased efficiency of energy conversion to photosynthesis; as in the case of experiments done with *Phaeodactylum tricornutum* (about 380 and 1000 ppmv CO_2_, [15]), *S. costatum* (350 and 1000 ppmv CO_2_, [14]), *Thalassiosira pseudonana* (∼390 to 750 ppmv CO_2_, [16])), *Asterionellopsis glacialis*, *Thalassiosira punctigera* and *Coscinodiscus wailesii* (from ∼20 to 800 ppmv [17]) under increasing CO_2_. Moreover, studies with natural diatom-rich phytoplankton communities have shown dominance of larger diatoms under enhanced CO_2_ concentrations in the Ross Sea [35] and Southern coast of Korea [36]. The positive response of diatoms is thought a consequence of an associated down-regulation of carbon concentrating mechanisms (CCMs) with increasing CO_2_ concentration (e.g. [37]–[38]), since the energy saved by a down-regulated CCM operation could be reallocated to carbon fixation and growth. Indeed both solitary (*Phaeodactylum tricornutum* and *Thalassiosira pseudonana*) and colony forming species (*S. costatum* and *Thalassiosira weissflogii*) have been shown to continue to increase cell division rates at CO_2_ levels higher than 600 µatm. In the present study the CO_2_ threshold isn't conclusive. However, the tendency line estimated suggest that *A. glacialis* cell division rates increased until ∼600 µatm CO_2_, potentially driven by the excess energy saved from the CCM, decreasing at CO_2_ values higher than 600 µatm probably related to low pH values. The apparent slightly higher sensitivity of *A. glacialis* to enhanced CO_2_ concentrations, for the CO_2_ treatments considered, might be species-specific, but an effect of the colony structure (shaped as star, zigzag and spiral) and the consequent proximity of sister cells cannot be excluded. Similar to other colonial phytoplantkon species, such as the cyanobacteria *Anabaena* sp. and *Nodularia spumigena* [39], the centre of *A. glacialis* colonies might have relatively high pH/low CO_2_ concentrations during the day in comparison to the bulk media, decreasing diffusive CO_2_ supply. Hence, the initial positive effect of increased CO_2_ availability, here more visible in photosynthesis (carbon production rate) than growth, was counterbalanced by the effects of the pH decrease. Increased chain length of *A. glacialis* may have influenced CO_2_ supply, but more importantly at more extreme conditions of pH exposure, may have maintained localised external pH closer to optimum. Finally, the modifications in colony length of *A. glacialis* might come as a compensation for a higher pH optimum (more alkaline) of this species, at the expense of energy and cell division rate. This may oppose the response of other species such as *Thalassiosira weissflogii* or *S. costatum* which form more linear colonies. In *Proboscia alata* cells formed spirals under the combined effect of low CO_2_ concentrations (below present concentrations and the range considered in this study) and high light [19]. However, in this case, the modification in morphology might be related to a strategy to reduce excess light penetration under low CO_2_ supply, thereby reducing reactive oxygen species production and keeping growth rate constant.

### Influence of carbonate chemistry speciation on chain length

Lower cell division rates found in this study were associated with longer chains of *A. glacialis*. In contrast, the growth rate of *S. costatum* has been found to be positively correlated with chain length both in cultures and enclosed natural communities [2]. Discrepancy in the correlation between colony growth and metabolic rates has been previously reported [7]. The increased chain length and proximity of the cells due to the observed colony structure under high CO_2_ concentrations might be a strategy to increase pH in the centre of the colonies during the light phase or may simply be a consequence of the nature of the bonds established between adjacent cells in a chain. These bonds vary from septa fusion [1] to attachment at the valve apices by exudation of polysaccharides [5] depending on the diatom species. Adjacent cells of *S. costatum* establish low flexibility bonds by connection of external tubes, which may break with increased turbulence. Under a low turbulence environment, as cell division rate rises, the number of cells in a given chain and time should increase independent of the type of cell-cell connections. *A. glacialis* cells bind by mucilage polysaccharide pads with high C:N and C:P [7]. However, no detectable increase in polysaccharide production was observed either by using Calcofluor White staining (data not shown) nor by a change in the C:P and C:N ratios under increasing CO_2_ concentrations/decreasing pH values. Therefore, longer chains under decreased cell division rates and slight turbulence due to mixing in this study might be connected to stronger bonds between polysaccharides at lower pH conditions. Interestingly, the elemental ratios found in this study were lower than Redfield and distinct from those found for the same species (different strain and growth conditions) by Burkhardt et al. [40], but within the range found in previous studies for cells (e.g. similar C:N to [16]) under nutrient replete conditions (C:P 27-135 and N:P 5-19, for a revision see [41]).

### Cell uptake/exudation balance

Under nutrient-replete conditions, lower cell division rates would be expected to be accompanied by increased cellular element quotas as observed here until 1470 µatm CO_2_. However, N, P and C quotas decreased with increasing CO_2_ from 1470 to 3400 µatm in spite of the decreasing trend in cell division rates. This is potentially due to increased exudation of dissolved organic compounds or variable nutrient uptake. Nutrient drawdown data is not conclusive (data not shown), but it is evident that P and Si uptake were higher at 3400 µatm CO_2_ concentrations than 600 µatm while nitrate showed no trend. Hence, the decrease of cellular quotas could not be explained by lower nutrient uptake. Similarly, in *Thalassiosira weissflogii* there wasn't a significant difference in Si uptake with increasing CO_2_ concentrations from ∼370 to 750 µatm, changing the rates of dissolution, efflux and incorporation into the frustule from ∼100 to 750 µatm [42]. Here, the difference between P, Si and nitrate drawdown may reflect a number of factors related to cell signalling (e.g. unsaturated aldehydes, see [43], [44]), energetics and membrane permeability.

Enhanced exudation of organic matter, namely carbohydrates, has been previously observed as a response to stressors such as increasing CO_2_ concentrations in coccolithophores [45] and nutrient limitation at the end of phytoplankton blooms [46]. Exudation as a response to low pH values might indeed explain the observed trend in carbon cellular quotas as depicted by uptake rates (DIC) that are greater than the accumulation rate of organic carbon. This is further supported by the POC production rate decrease with the increase of dissolved organic exudation rates at higher CO_2_ levels.

### Summary and conclusions

The present study shows that cell division rates of *A. glacialis* did not change significantly from 320 to 750 µatm of pCO_2_, but started to decrease towards higher CO_2_ levels. This decrease was accompanied by an increase in cellular element quotas and organic matter production rates until 1470 µatm, and by increased DOC exudation at CO_2_ levels higher than that, with no changes in stoichiometric element ratios. Moreover, the relative number of cells per chain (chain length) increased at elevated CO_2_, potentially limiting nutrient diffusion under deplete conditions. Longer chains and modified chain morphology could influence buoyancy and sinking rates as in the case of other species [3], [4], [47]. If *A. glacialis* follows the response of *S. costatum* [4] the increased buoyancy with chain length could in turn positively affect growth in the natural environment since cells closer to the surface of the ocean will be exposed to an increased average light intensity. Hence, the chain formation strategy (i.e. longer chains) displayed by *A. glacialis* might be advantageous under future scenarios of elevated CO_2_ where increased light supply might further increase photosynthesis. Depending on the sensitivity of co-occurring species, these changes could affect the plankton community composition. Finally, the increased exudation of dissolved organic carbon might increase aggregation and potential for sinking of particles.
